# Anti-Hepatitis C Virus Activity of Uridine Derivatives of 2-Deoxy Sugars

**DOI:** 10.3390/molecules23071547

**Published:** 2018-06-27

**Authors:** Ewelina Krol, Ilona Wandzik, Gabriela Pastuch-Gawolek, Boguslaw Szewczyk

**Affiliations:** 1 Department of Recombinant Vaccines, Intercollegiate Faculty of Biotechnology, University of Gdansk and Medical University of Gdansk, Abrahama 58, 80-307 Gdansk, Poland; szewczyk@biotech.ug.gda.pl; 2 Department of Organic Chemistry, Bioorganic Chemistry and Biotechnology, Faculty of Chemistry, Silesian University of Technology, Krzywoustego 4, 44-100 Gliwice, Poland; Ilona.Wandzik@polsl.pl (I.W.); gabriela.pastuch@polsl.pl (G.P.-G.); 3 Biotechnology Center, Silesian University of Technology, Krzywoustego 8, 44-100 Gliwice, Poland

**Keywords:** hepatitis C virus, antiviral compounds, uridine, 2-deoxy sugars, glycosylation inhibition

## Abstract

Hepatitis C virus (HCV), the etiological agent of the most common and dangerous diseases of the liver, is a major health problem worldwide. Despite many attempts, there is still no vaccine available. Although many drugs have been approved for use mostly in combination regimen, their high costs make them out of reach in less developed regions. Previously, we have synthesized a series of compounds belonging to uridine derivatives of 2-deoxy sugars and have proved that some of them possess antiviral activity against influenza A virus associated with N-glycosylation inhibition. Here, we analyze the antiviral properties of these compounds against HCV. Using cell culture-derived HCV (HCVcc), HCV pseudoparticles (HCVpp), and replicon cell lines, we have shown high anti-HCV activity of two compounds. Our results indicated that compounds **2** and **4** significantly reduced HCVcc propagation with IC_50_ values in low μM range. Further experiments using the HCVpp system confirmed that both compounds significantly impaired the infectivity of produced HCVpp due to the inhibition of the correct maturation of viral glycoproteins. Overall, our results suggest that inhibiting the glycosylation process might be a good target for new therapeutics not only against HCV, but other important viral pathogens which contain envelopes with highly glycosylated proteins.

## 1. Introduction

Hepatitis C virus (HCV) belongs to the *Hepacivirus* genus in the *Flaviviridae* family. It is a positive-stranded RNA virus which is the major cause of chronic liver diseases, like cirrhosis and liver carcinoma [1]. As the result, HCV is the main factor responsible for liver transplantation worldwide, which makes the virus one of the most serious burdens to public health. Until 2011, a combination of pegylated IFN alpha and ribavirin was used in the treatment of HCV-infected patients [2]. However, it was ineffective in at least 50% of cases and was associated with numerous side-effects. Significant effort has been made to search for new and more effective treatments. The most intensive research has been focused on chemically-modified nucleoside and nucleotide derivatives as effective anti-HCV agents [3,4]. A highly active drug, sofosbuvir, belonging to the nucleotide prodrugs, is one of the most successful examples [5,6].

The introduction of highly effective drugs like boceprevir, telaprevir, sofosbuvir, ledipasvir, daclatasvir, and simeprevir has resulted in higher cure rates and shortening the duration of treatment. These drugs belong to direct-acting antivirals (DAAs), inhibiting the non-structural proteins crucial for virus replication such as NS3/4A proteases, NS5A or NS5B polymerase, However, the clinical usage of the drugs has been associated with the emergence of rapid viral resistance [7,8,9]. The combinations of DAAs in interferon-free regimen eliminated troublesome side effects caused by interferons. However, it is prone to drug-drug interactions. FDA-approved combinations of drugs include Harvoni, a combination of sofosbuvir and ledipasvir, Epclusa (sofosbuvir and velpatasvir), Viekira Pak (ombitasvir, paritaprevir, ritonavir and dasabuvir), Mavyret (glecaprevir and pibrentasvir), and Zepatier (elbasvir and grazoprevir) [10]. Although, current drug combinations are well tolerated, their use is limited by extremely high cost restricting the access to therapy which is low on a global scale [9,11]. Thus, the development of new anti-HCV compounds targeting different steps of the HCV life cycle is required to prevent the emergence of drug-resistant viral mutants and to increase the effectiveness and availability of potential therapy. 

HCV envelope glycoproteins E1 and E2, abundant on the viral surface, form a heterodimer which plays an important role in viral entry, fusion, and secretion [12]. Moreover, E1/E2 heterodimer is the major target for virus-neutralizing antibodies [13,14]. E1 and E2 are highly glycosylated proteins, with glycans amounting to about 50% of the heterodimer mass [15]. Host chaperone proteins like calnexin and BIP are involved in the addition of sugar groups during the folding of these two proteins. E1 and E2 have five to six and nine to eleven potential N-glycosylation sites, respectively [16]. Their number depends on the virus genotype. All N-glycosylation sites of E2 are highly conserved. Glycans are essential for proper folding and activity of many viral glycoproteins. The removal of *N*-oligosaccharides provokes protein misfolding, leading to aggregation and retention in the endoplasmic reticulum or proteasome degradation [17,18]. The intensive research has been performed to define the role of N-glycans present on both HCV glycoproteins. These findings have ruled out the hypothesis that glycosylation on residues 196 and 305 of E1 are crucial for E1–E2 heterodimerization [19]. Moreover, the lack of sugars on N-glycosylation sites of E1 can influence the interaction with calnexin [20]. For E2 glycoprotein it was shown that N1, N8, and N10 glycosylation sites are crucial for correct folding and heterodimerization. Furthermore, glycans in position N7 have a vital role in viral entry, and in position N1, N2, N4, N6, or N11 contribute to immune evasion [21].

Some steps of the HCV morphogenesis can serve for a choice of potential targets for antiviral drugs. Additionally, the targeting of host cellular functions required for virus replication can constitute an alternative approach for anti-HCV therapy [22]. Additionally, due to the low genetic variability of host factors, host-targeting agents (HTAs) can exhibit antiviral activity against many HCV viral genotypes [23]. Combination therapy based on DAAs and HTAs could be a promising option for HCV treatment in future. 

**Figure 1:**
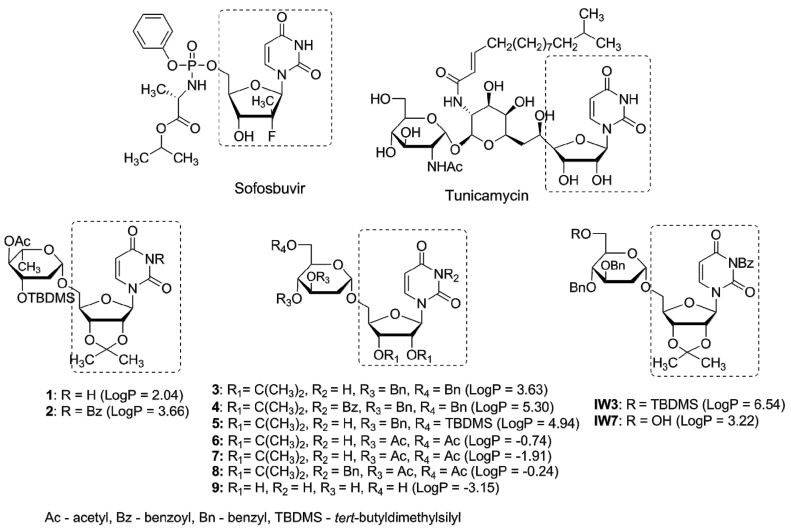
Nucleotide antiviral agents sofosbuvir and tunicamycin and uridine derivatives of 2-deoxy sugars: **1**–**9**, **IW3**, **IW7**. LogP values calculated with the XLogP3 algorithm [27] are presented in brackets.

Glycosyltransferases (GTs) are a large class of enzymes participating among others in maturation of viral glycoproteins during glycosylation process. The arrest or alternation of the glycosylation process of viral proteins by effective inhibitors of GTs usually results in antiviral effects. As an example, tunicamycin, a nucleoside antibiotic containing uridine and 11-carbon disaccharide tunicamine in its structure inhibits N-glycosylation in eukaryotes. Inspired by tunicamycin activity we have recently synthesized a small library of uridine derivatives of 2-deoxy sugars containing hydrophobic motifs in their structures (compounds **1**–**9**, **IW3**, **IW7**, Figure 1) [24,25,26]. Lipophilic moieties were included because the common approach in antiviral prodrug design is to increase passive permeability and several antiviral drugs, e.g., sofosbuvir or oseltamivir are administered in the form of more lipophilic prodrugs in order to increase permeability. Uridine derivatives synthesized by us turned out to be active in vitro against classical swine fever virus (CSFV), a member of the family *Flaviviridae* [24], and influenza virus, belonging to the *Orthomyxoviridae* [25]. The observed antiviral potency was attributed to impaired maturation of viral proteins caused by the inhibition of N-glycosylation process in cis-Golgi or very early in medial-Golgi compartments. Moreover an analysis of physicochemical properties of studied compounds demonstrated a significant correlation between lipophilicity and antiviral activity, the most lipophilic were the most active [26]. 

On the basis of our previous results, the aim of this work was to analyze the antiviral activity of uridine derivatives of 2-deoxy sugars (compounds **1**–**9**) on HCV infectivity and morphogenesis. We have shown that two of the tested compounds, **2** and **4**, show high antiviral activity against HCV. Both compounds significantly reduced the propagation of infectious HCV virus in cell culture (HCVcc) affecting the maturation of viral glycoproteins which was confirmed in pseudoparticles (HCVpp) system when the infectivity of HCVpp was reduced due to the incorporation of altered forms of glycoproteins into these pseudoparticles.

## 2. Results

### 2.1. Anti-HCV Activity of 2-Deoxy Sugar Derivatives of Uridine (HCVcc) 

**Figure 2:**
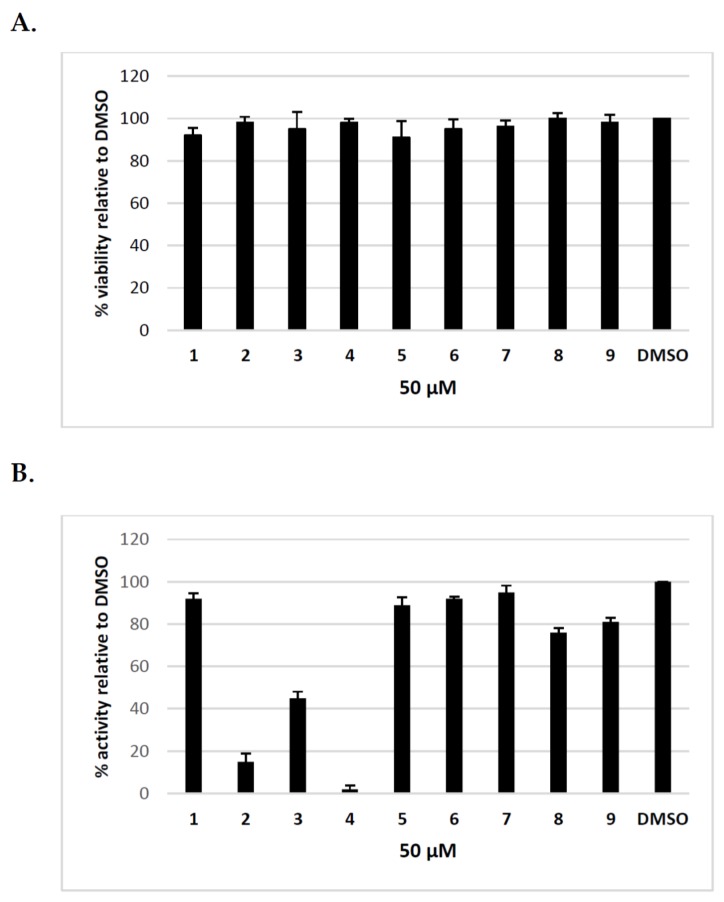
The inhibitory effect of uridine derivatives of 2-deoxy sugars (compounds **1**–**9**) against hepatitis C virus. (**A**) Cell viability of Huh-7.5 cells after treatment for 72 h with compounds **1**–**9** using MTS assay; (**B**) The anti-HCVcc antiviral activity of compounds **1**–**9** (50 μM). Huh-7.5 cells were infected with HCVcc at an MOI of 0.1, treated with tested compounds (50 μM) for 72 h and IPMA assay was performed on fixed cells to visualize pseudo-plaques. Plaques were counted and presented as % in comparison to the number in DMSO-treated cells expressed as 100%. Error bars indicate standard deviations from three experiments.

**Figure 3:**
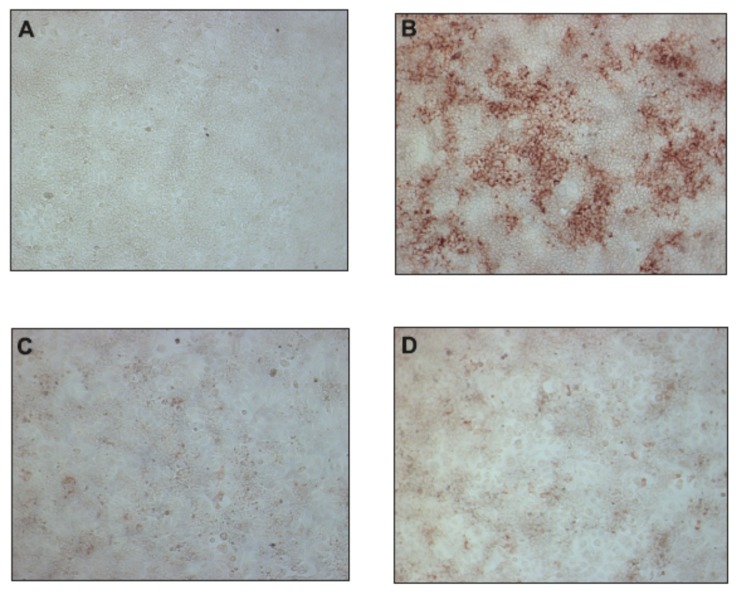
The effect of uridine derivatives of 2-deoxy sugars (compounds **2** and **4**) on HCV cc pseudo-plaque formation. Huh-7.5 cells were infected with HCVcc at an MOI of 0.1 (**B**–**D**) or mock infected (**A**). At 3 h p.i., the virus was removed and the fresh medium with 50 μM of compound **2** (**C**) or **4** (**D**) was added. After three days p.i., cells were fixed and pseudo-plaques were detected by immunostaining with anti-core antibody.

Previously, we have shown that uridine derivatives of 2-deoxy sugars exert good anti-classical swine fever virus and anti-influenza virus activity [24,25,26]. In this study, we evaluated the in vitro activities of these compounds against hepatitis C virus. Initially, we examined the cytotoxicity of synthesized compounds in Huh-7.5 cells using MTS assay. No toxicity to Huh-7.5 cells at a concentration of 50 μM was observed (Figure 2A). The calculated CC_50_ values for compounds **1**–**9** were as follows: 85, 119, 89, 151, 83, 210, 258, 223, 245 μM. To evaluate the anti-HCV activity of all compounds, Huh-7.5 cell culture-derived HCV (HCVcc), which allows for complete HCV life cycle in cell culture including in vitro production and secretion of HCVcc, was used [28,29]. Jc1/JFH genotype 2a infected Huh-7.5 cells were treated with 50 μM of inhibitors or DMSO and HCVcc pseudoplaque reduction assay was performed as the preliminary screening of compounds as previously described [30]. The antiviral activity was evaluated by the reduction of infected cells (pseudoplaques). Viral pseudoplaque formation was detected by immunostaining the core protein in HCV infected Huh-7.5 cells grown in the presence of tested compounds. As shown in Figure 2B compounds **3**,**8** and **9** showed minor anti-HCV activity, whereas compounds **1**,**5**,**6**, and **7** showed no obvious anti-HCV activity. However, the results demonstrated that compounds **2** and **4** displayed high antiviral activity. These compounds nearly completely inhibited HCV virus infection relative to DMSO-treated cells at tested concentration. The exemplary results of IPMA assay where a significant reduction in size and number of positive infected foci was observed in comparison to a positive control are shown in Figure 3. The obtained results were in agreement with previously published results using influenza A virus, which confirmed their antiviral potential. 

**Figure 4:**
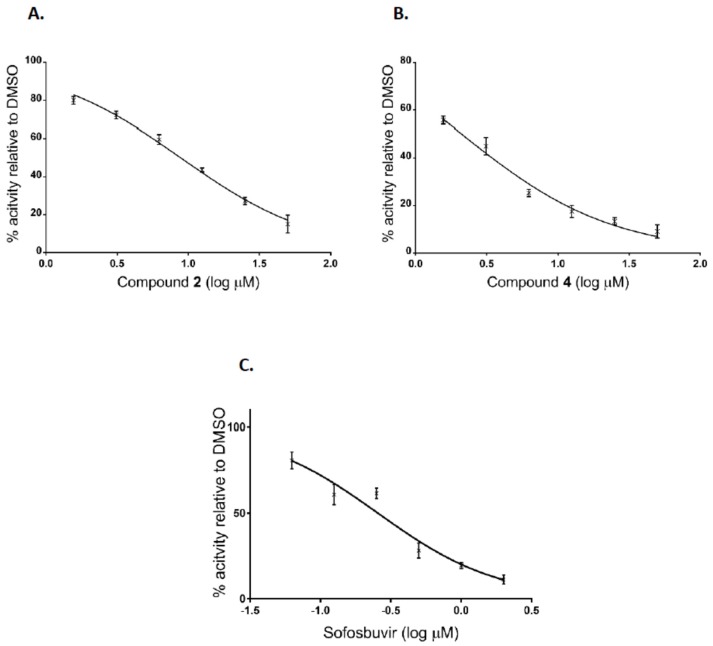
Inhibition curves of uridine derivatives of 2-deoxy sugars (compounds **2** and **4**) and sofosbuvir. HCV infected Huh7-J20 cells were incubated for 72 h in the presence of varying concentrations of compound **2** (**A**), **4** (**B**), or sofosbuvir (**C**) and their effect on viral replication was evaluated by SEAP assay from the culture supernatant. Each concentration was tested in triplicate and the results are expressed as the mean and standard deviation.

Additionally, after the preliminary screening at 50 μM, dose-response assays were performed for two of the most active compounds to calculate IC_50_ values. The experiments were conducted using human hepatoma cell line Huh7-J20, which stably expresses EGFP fused in-frame to secretory alkaline phosphatase (SEAP) via a recognition sequence of the viral NS3/4A serine protease as a reporter system [31]. The SEAP level in the culture medium of HCVcc infected cells directly correlates with the level of viral replication. Overnight grown Huh7-J20 cells infected with HCVcc at multiplicity of infection (MOI) of 0.1 were incubated for 72 h in the presence of varying, nontoxic as determined by MTS assay concentrations of inhibitors and their effect on viral replication was evaluated by SEAP assay from the culture supernatant. Sofosbuvir, an inhibitor of the NS5B RNA-dependent RNA polymerase was used as a positive control [5,6]. Compounds **2** and **4** showed significant reduction in the SEAP level indicating high anti-HCV activity with IC_50_ values of 8.9 and 2.1 μM, respectively (Figure 4A,B). Both compounds exhibited low cytotoxicity (CC_50_ values of 113 and 142 μM) resulting in selectivity indexes (SI), defined as the CC_50_/IC_50_ ratio of 12.7 and 67.6. Therefore, these two compounds were selected for further evaluation. Sofosbuvir, a positive control in the experiment, showed IC_50_ value of 0.26 μM and calculated SI of about 120 (Figure 4C). 

### 2.2. Uridine Derivatives of 2-Deoxy Sugars Do Not Target the HCV Replication Process

**Figure 5:**
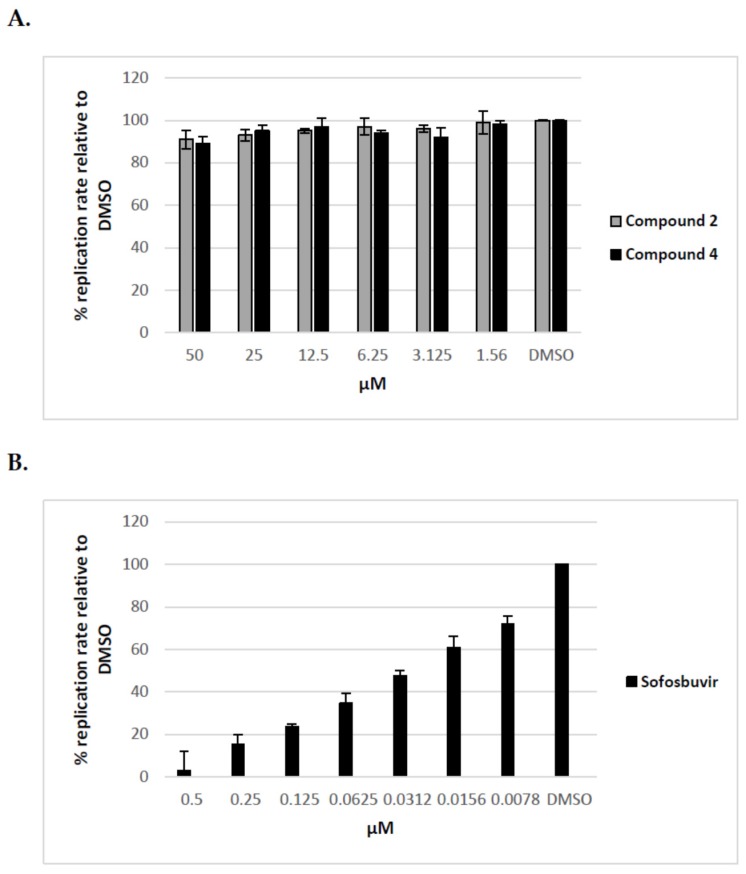
The influence of uridine derivatives of 2-deoxy sugars and sofosbuvir on HCV replication. Huh7-J17 cells were plated together with serial dilutions of compound **2** and **4** (**A**) or sofosbuvir (**B**) for 72 h followed by luciferase activity measurement in cell lysates. DMSO-treated cells are expressed as 100%. Error bars indicate standard deviations from three experiments.

Our previous results indicated that the antiviral activity of uridine derivatives of 2-deoxy sugars is not associated with the replication process. To confirm this for HCV, the ability of the compounds to inhibit HCV replication has been checked using Huh7-J17 stable cell line. This cell line expresses monocistronic replicon encoding non-structural proteins, structural core protein and firefly luciferase, the levels of which directly correlate with virus RNA replication [32]. Cells were seeded together with different doses of compounds **2** and **4** and the level of RNA replication was estimated 72 h later by the luciferase levels measurement. Sofosbuvir, an established HCV replication inhibitor was used as a positive control. The cytotoxicity of compounds was determined using MTS assay. As expected, our results confirmed that tested compounds have no effect on HCV replication process, as no differences in the level of luciferase activity were detected in comparison to the control (Figure 5A). Sofosbuvir showed high inhibition of the viral replicon RNA with calculated IC_50_ value of 0.028 μM (Figure 5B). 

### 2.3. Effect of Uridine Derivatives of 2-Deoxy Sugars on HCVpp Infectivity

**Figure 6:**
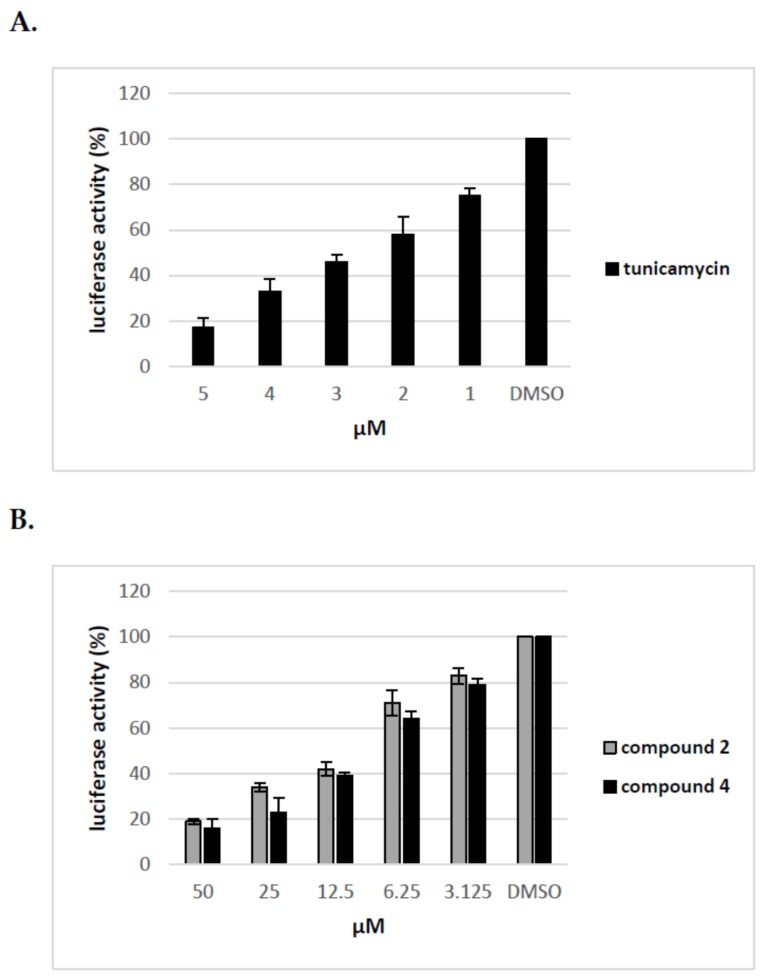
The effect of tunicamycin and uridine derivatives of 2-deoxy sugars on HCVpp secretion and/or infectivity. HEK-293 T cells were transfected with three plasmids to produce HCVpp. 16 h p.t. fresh medium with different doses of tunicamycin (**A**) or compounds **2** and **4** (**B**) were added. Cell culture supernatants containing pseudoparticles were harvested 48 h post transfection and used to infect overnight Huh-7.5 cells. Infection efficiency after 72 h was measured by luciferase assay. The infectivity of HCVpp produced in DMSO-treated cells are expressed as 100%. Error bars indicate standard deviations from three experiments.

We have previously shown that uridine derivatives of 2-deoxy sugars show antiviral activity by targeting the glycan processing steps during maturation of viral glycoproteins. We have also demonstrated that, in the case of viruses belonging to *Flaviviridae* or *Orthomyxoviridea* families, glycoproteins lacking oligosaccharides due to glycosylation inhibitory treatment are most probably rapidly degraded in host cells and cannot be detected [24,25,26]. To further test the effect of compounds on maturation of HCV viral glycoproteins the surrogate retrovirus-based pseudoparticle system (HCVpp) was used [33,34]. This system, which enables the production and secretion of HCVpp particles, consists of retroviral particles expressing HCV E1 and E2 glycoproteins on the surface and firefly luciferase or GFP as a reporter gene to allow quantitate measurement of HCV entry into target cells. HCVpp produced by co-transfection of HEK-293T with three expression plasmids in the presence of various concentrations of compounds for 72 h were used to infect Huh-7.5 cells. Tunicamycin, which prevents protein N-glycosylation, was used as a control. In parallel, MTS cell viability assay was performed which showed that no toxicity was observed with all doses of compounds used in the experiment. As shown in Figure 6 compounds **2** and **4** demonstrated a significant, dose-dependent reduction in HCVpp production and/or infectivity as measured by luciferase activity in the lysates of Huh-7.5 infected cells. We found that after treatment with 50 μM of compounds **2** and **4** the HCVpp production and/or infectivity was reduced in about 80% relative to DMSO control. The reduced HCVpp infectivity was also shown for tunicamycin, a positive control used in the experiment. Tunicamycin at a concentration of 5 μM exhibited more than 80% inhibition of infection. The decrease in infectivity of pseudoparticles as a result of inhibitory treatment may be associated with impaired incorporation of E1E2 glycoproteins into pseudoparticles or incorporation of altered forms of E1E2 proteins that have no ability to bind receptors during infection. 

### 2.4. Uridine Derivatives of 2-Deoxy Sugars (Compounds 2 and 4) Impairs Only the Production of HCVpp 

**Figure 7:**
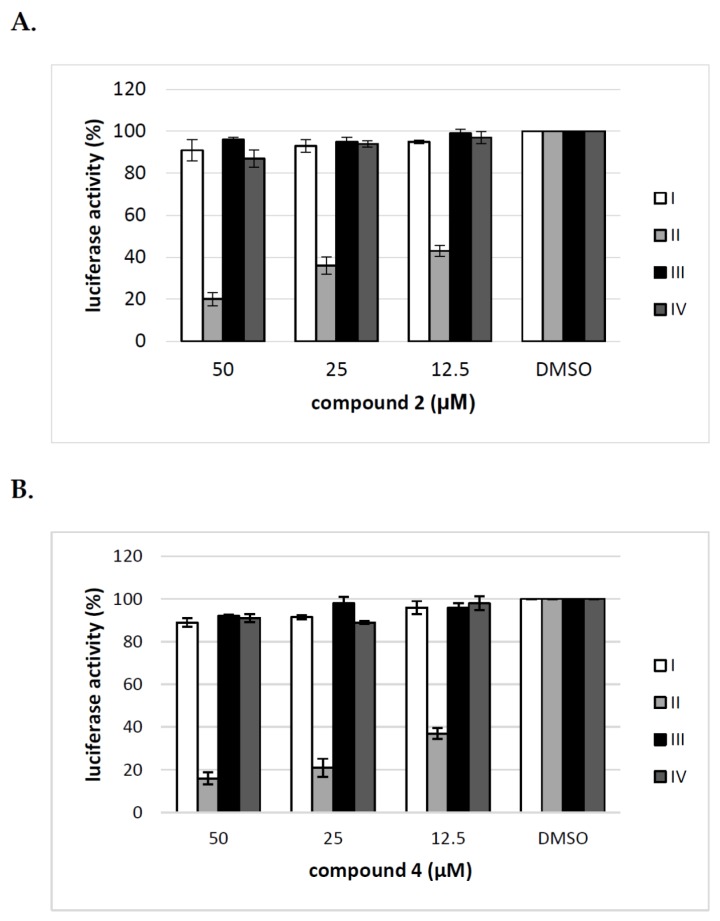
Analysis of the effect of compound **2** and **4** on different stages of HCVpp production. Compound **2** (**A**) and **4** (**B**) were added to: HEK-293T cells o/n before transfection (I), HEK-293T cells 16 h post-transfection (II), Huh-7.5 cells o/n before infection (III), Huh-7.5 cells for 5 h during infection (IV). Infection efficiency after 72 h was measured by luciferase assay. The infectivity of HCVpp produced in DMSO-treated cells are expressed as 100%. Error bars indicate standard deviations from three experiments.

To elucidate further the mechanism of action of tested compounds, we added compound **2** and **4** at different time points during HCVpp production and infection of Huh-7.5 cells and calculated the inhibitory activity of compounds at each time point. The compounds were added: before transfection of HEK293T cells (I), 16 h post transfection of HEK293T cells (II), before infection of Huh-7.5 cells (III), during infection of Huh-7.5 cells for 72 h (IV). The obtained results from three independent experiments presented in Figure 7 clearly showed that the significant decrease in infectivity of HCVpp is observed when the inhibitor is added to HEK293T cells only after transfection, not before or during HCVpp infection of Huh-7.5 cells. These results suggest that the pseudoparticles generated during the treatment of HEK293T cells with tested inhibitors may be less infectious probably due to changes in glycosylation status or they are produced in lower amounts. 

### 2.5. Effect of Uridine Derivatives of 2-Deoxy Sugars on HCV Glycoprotein Accumulation in HEK293T Cells

**Figure 8:**
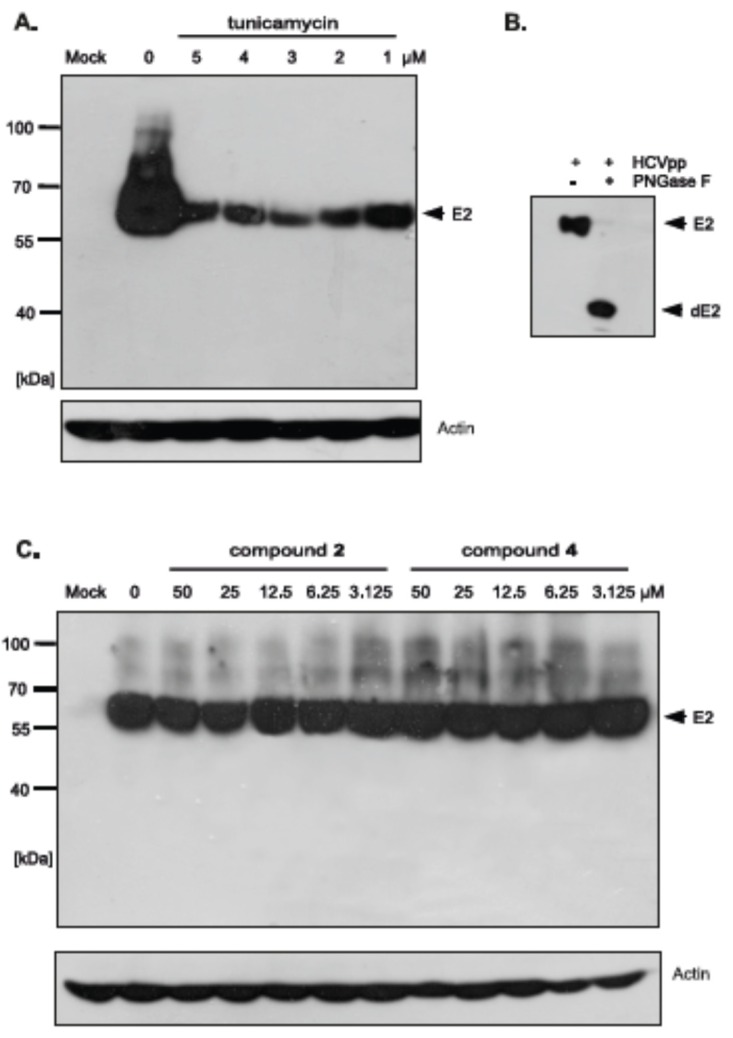
The effect of tunicamycin and uridine derivatives of 2-deoxy sugars on E2 protein accumulation in HEK-293T cells. HEK-293 T cells were transfected with three plasmids to produce HCVpp. 16 h post transfection fresh medium with different doses of tunicamycin (**A**) or compounds **2** and **4** (**C**) were added. After 48 h post-transfection cells were lysed and proteins were separated by SDS–PAGE. Additionally, cell lysates were first digested with peptide:N-glycosidase F (PNGase F) followed by SDS-PAGE (**B**). Western blot analysis was performed using the specific anti-E2 MAb (AP33) and anti-actin monoclonal antibody.

The differences in HCVpp production and/or infectivity after treatment with different compounds belonging to uridine derivatives of 2-deoxy sugars prompted us to analyze the influence of compounds **2** and **4** on HCV E1, E2 production and accumulation in HEK293T, because these proteins are further incorporated to mature HCVpp particles. They are later used in Huh-7.5 cells infection. Transfected HEK293T cells treated with tunicamycin were used as a positive control. Western blotting analysis showed that a significant disruption in the accumulation of E2 glycoprotein was observed after treatment with all tested doses of tunicamycin (Figure 8A). It is worth emphasizing that no unglycosylated forms of E2 glycoprotein were detected after tunicamycin treatment. The E2 glycoprotein synthesized in HEK293T cells without the addition of tunicamycin and deglycosylated enzymatically with PNGase F was detected in Western blot using the same antibody, which confirmed that the lack of unglycosylated forms of E protein after tunicamycin treatment was caused by the lack of protein and not due to the loss of the epitope responsible for antibody reactivity (Figure 8B). In case of compound **2** and **4** treatment, no change in the accumulation was observed, which suggests that the level of viral glycoprotein synthesis is not affected by these compounds (Figure 8C). Therefore, it could be concluded that the differences in HCVpp infectivity may be caused by the incorporation of modified forms of glycoproteins or a reduction in the incorporation of glycoproteins into the pseudoparticles. 

### 2.6. Uridine Derivatives of 2-Deoxy Sugars Change the Incorporation of Glycoproteins into HCVpp

**Figure 9:**
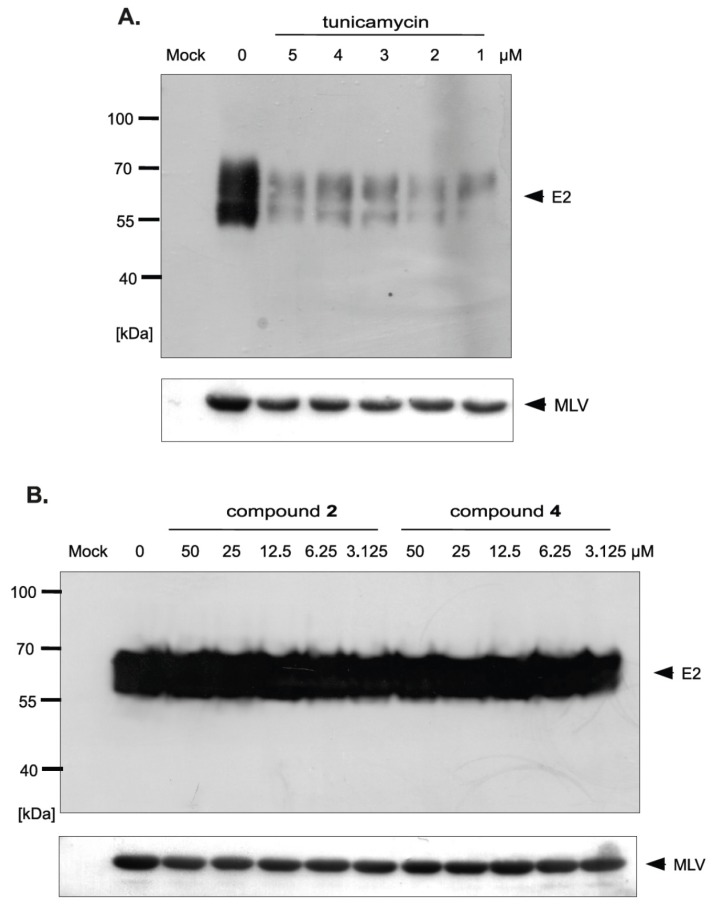
The effect of tunicamycin and compounds **2** and **4** on E2 protein accumulation in HCVpp and the amount of HCVpp in concentrated medium from HEK293T cells 48 h after transfection. HEK-293 T cells were transfected with three plasmids to produce HCVpp. 16 h p.t. fresh medium with different doses of tunicamycin (**A**) or compounds **2** and **4** (**B**) were added. After 48 h post transfection HCVpp were harvested from the medium, concentrated by ultracentrifugation, subjected to SDS-PAGE and analyzed by Western blot using anti-E2 MAb (AP33) and anti-MLV capsid antibody.

In order to investigate whether there is a difference in the incorporation of glycoproteins into pseudoparticles generated in the presence of inhibitors, the purification and concentration of viral particles present in the culture medium from HEK293T cells producing HCVpp was performed. The supernatants of transfected HEK293T cells treated with tunicamycin or increasing amounts of tested inhibitors were concentrated 100× by ultracentrifugation on a 20% sucrose cushion and analyzed by Western blotting. The obtained results indicated that, in the case of tunicamycin, there was a significant reduction in the amount of E2 glycoprotein incorporated into HCVpp (Figure 9A). However, after treatment with compound **2** and **4** no reduction in the amount of the protein was observed, which suggested that probably after the treatment the modified forms of E2 are incorporated into the pseudopraticles (Figure 9B). 

Moreover, the Western blot analysis of concentrated HCVpp using anti-capsid MLV antibody was also performed. The analysis showed that no changes in the amount of MLV particles were observed after tunicamycin (Figure 9A) as well as compound **2** and **4** treatment (Figure 9B) in comparison to the positive control (HCVpp from HEK293T cells produced without inhibitors). These results were in agreement with previously published results that MLV core can be secreted as ‘bald’ particles without incorporation of viral envelope glycoproteins [33]. The results obtained in this experiment may indicate that, although the amount of pseudoparticles does not change, the resulting pseudoparticles contain no glycoproteins incorporated into the envelope in case of tunicamycin treatment or modified forms of E2 in case of treatment with compound **2** and **4**, which have reduced ability to bind the receptors, thus directly effecting HCVpp infectivity. 

## 3. Discussion

DAA-based combination regimens for HCV treatment have been shown to be highly effective and well-tolerated. However, due to extremely high costs they are often beyond accessibility. Shortening the duration of treatment could greatly reduce the costs and improve the access to anti-HCV therapy. Due to these reasons new therapeutic options are still needed. The process of HCV morphogenesis can be a possible target for a new generation of antiviral drugs. Viral glycoproteins are an important component of virions and participate in the entry process during viral infection by binding to specific receptors present on the surface of target cells and inducing fusions between the viral envelope and the cell membrane. In addition, they participate in the assembly of progeny viral particles and modulate the immune response. 

The maturation of the surface viral glycoproteins depends on the host endoplasmic reticulum (ER) protein-folding machinery to form the proper three-dimensional structure needed for their activity. The inhibition of ER-folding process usually results in prevention of proper folding of viral glycoproteins which are not incorporated into the mature viral particles. The N-glycosylation process plays a vital role in the maturation of many viral proteins (stability and correct folding), as well as in conferring resistance to protein degradation and recognition by the immune system [17,18,35]. There are many natural and chemically-synthesized glycosylation inhibitors with potent antiviral effects [36,37,38,39,40]. Antibiotic tunicamycin is one of them [41,42]. We have previously reported the antiviral activity of compounds **IW3** and **IW7** against classical swine fever virus and influenza A virus [24,25]. The detailed studies on the antiviral properties and mechanism of action of these compounds revealed that they belong to N-glycosylation inhibitors targeting the late step of this process. We have further synthesized a series of **IW3** and **IW7** analogues (compounds **1**–**9**) which possess different structural modifications in 2-deoxy sugar or uridine parts in comparison to **IW3** and **IW7** in order to increase the antiviral activity. All of compounds (**1**–**9**) were previously examined against influenza A virus [26]. Among synthesized compounds, compounds **2** and **4** have been found to be the most active. 

In this study, the in vitro antiviral properties of uridine derivatives of 2-deoxy sugars **1–9** were evaluated against hepatitis C virus. To provide further evidence of the potential antiviral activity of synthesized compounds we used different models of hepatitis C virus: HCVcc, replicon cell line and HCVpp. The HCVcc system was developed to study the complete HCV life cycle in cell culture, which greatly enriched the knowledge about HCV biology [28,29]. Our studies using HCVcc system indicated that two compounds **2** and **4** containing hydrophobic motifs in their structures significantly inhibited HCV infection at a non-cytotoxic concentration (Figure 2). Compounds **2** and **4** exhibited potent anti-HCV activities with IC_50_ values of 8.9 and 2.1 μM, respectively showing their potential. Using Huh7-J17 replicon stable cell line coding only for all non-structural proteins we have also excluded the influence of compounds **2** and **4** on HCV replication process (Figure 5) [32]. Due to the lack of structural proteins, no infectious particles are produced in these cells so there is no possibility for antiviral screening of compounds targeting viral glycoproteins using this system. 

Extensive research has been conducted for antiviral screening of novel compounds using HCVpp system [43,44,45,46,47,48]. This system allows for studying the role of glycoproteins in HCV entry, to identify receptors important during attachment process or to gain the important knowledge about the internalization process [49]. HCV pseudo-particles were among others used to study the activity of glucosidase inhibitors on HCVpp infectivity. It was demonstrated that the reduction in HCVpp infectivity was due to incorporation of misfolded glycoproteins into the particles [50]. This model was used for the confirmation of the antiviral properties of compounds **2** and **4**. After treatment with these compounds, the HCVpp infectivity was significantly decreased (Figure 6B). We have shown that although compounds **2** and **4** do not change the production of HCVpp, because the number of HCVpp was not decreased (Figure 9B), they probably change their binding properties (Figure 6B), which directly correlates with the reduction of interaction with Huh-7.5 cells. Moreover, we demonstrated that the amount of glycoproteins produced in HEK293T cells (Figure 8C) and incorporated to HCVpp was also not affected (Figure 9B). These results differ from those obtained with classical swine fever virus and influenza virus where it was shown that misfolded proteins after uridine derivatives of 2-deoxy sugars treatment were degraded and did not accumulate in CSFV- and MDCK-infected cells [24,25,26]. Overall, our data have shown that the reduction of HCVpp infectivity is probably caused by the incorporation of modified forms of envelope glycoproteins into the pseudoparticles. These results are in agreement with previously-published data when testing the derivatives of deoxynojirimycin iminosugars (inhibitors of endoplasmic reticulum α-glucosidases I and II) as potential antiviral compounds [50].

It is worth noting that, in the case of tunicamycin, the reduction of HCVpp infectivity was caused by impaired incorporation of glycoproteins into HCVpp. We demonstrated that the amount of glycoprotein E2 was significantly decreased after tunicamycin treatment both in HEK293T cells (Figure 8A) as well as in concentrated HCVpp (Figure 9A), however, the amount of bald MLV particles was constant in comparison to the control (Figure 9A). The decreased amount of modified glycoproteins after tunicamycin treatment may be caused by quick degradation of incorrectly matured proteins. We hypothesize that changes of N-glycosylation profile of HCV glycoproteins and their quick ER-associated degradation process lead to reduced availability of these proteins during HCVpp morphogenesis. However, the lack of degradation of proteins after treatment with compound **2** and **4** was unexpected. 

The possible explanation of this phenomenon is that HCVpp model may not be the optimum solution to study HCV morphogenesis and the production of envelope glycoproteins incorporated into pseudoparticles. Previously, the differences in the glycosylation of glycoproteins obtained in HCVpp and HCVcc systems have been shown [51]. The differences in glycosylation may be due to the fact that the assembly of HCVcc occurs in ER-derived compartments and is associated with the presence of low-density lipoproteins [52,53], whereas the assembly of HCVpp molecules occurs in post-Golgi compartments [54]. This suggests that in these two systems, the glycans associated with viral glycoproteins are not treated in the same way with the glycosidases and glycosyltransferases present in the Golgi apparatus. Moreover, HCVpp are not an optimum model to study the glycosylation process because they are produced in kidney cell line (HEK293T), a cell line that is not permissive to HCV replication, while HCV infects mainly hepatocytes [55]. These facts may account for lack of degradation of modified glycoproteins after treatment with compounds **2** and **4** which, we hypothesize, may possess the mechanism of action similar to **IW3** and **IW7** compounds belonging to the inhibitors of late steps of glycosylation process in contrast to tunicamycin, an inhibitor of the first step of N-glycosylation. However, further studies are needed to confirm that the mechanism underlying the antiviral activity of compounds **2** and **4** is similar to this observed for **IW3** and **IW7** compounds. Experiments using unrelated viruses e.g., herpeviruses, for which glycan-free polypeptides might be more stable than for HCV and can be detected by immunoblotting, would throw more light on compound **2** and **4** activity on glycans removal or their modifications. 

In order to evaluate the influence of lipophilic moieties contained in compounds **1**–**9** on antiviral activity, lipophilicity parameter LogP was calculated and confronted with the antiviral activity. Recently we have demonstrated a good agreement between experimental data and theoretical logP values for several uridine derivatives [56]. A good agreement was obtained when XLogP3 algorithm [27] was applied, thus in this study XlogP3 have been calculated for structures **1**–**9** (Figure 1). It was found that the most active compounds against hepatitis C virus were the most lipophilic compounds **2** and **4** (LogP 3.66 and 5.30, respectively). In addition to lipophilicity, the presence of benzoyl group at N3 of the uracil part was equally important. This observation is consistent with our previous results concerning the ability to inhibit classical swine fever virus [24] and influenza A virus [25]. Again, the most active compounds **IW3** and **IW7** are, at the same time, very lipophilic (LogP 6.54 and 3.22 respectively) and contain a benzoyl group at N3 of the uracil part.

In summary, we have demonstrated the antiviral activity of uridine derivatives of 2-deoxy sugars against hepatitis C virus. Previously, we have shown that this class of compounds can inhibit classical swine fever virus and influenza A virus propagation in cell cultures [24,25,26]. The current findings using hepatitis C virus have confirmed their broad-range antiviral activity. We have proved that the mechanism underlying the antiviral activity of compounds described in this paper may be universal and they can be employed to control other viral infections. Further modifications of compounds **2** and **4** to improve their activity are continued in our laboratory. 

## 4. Materials and Methods 

### 4.1. Antiviral Compounds, Cells and Viruses

The compounds were synthesized as described previously [26,57,58]. The stock solutions were prepared in DMSO and stored in −20 °C until future use. Sofosbuvir was purchased from Selleckchem (Munich, Germany) and tunicamycin was purchased from Sigma-Aldrich (Saint Louis, MO, USA). 

Human epithelial kidney cells (HEK293T) and human hepatoma cells Huh-7.5 were grown in Dulbecco’s Modified Eagle Medium (DMEM) (Sigma-Aldrich), containing 10% fetal bovine serum, 0.5 mM GlutaMax (Invitrogen, Carlsbad, CA, USA) and 100 U/mL penicillin/mL and 100 mg/mL streptomycin at 37 °C under 5% CO_2_. Another variant of the human hepatoma cell line, Huh7-J20, which is stably transformed with the secreted alkaline phosphatase (SEAP) reporter system under HCV promoter [34] and replicon Huh7-J17, which stably expresses viral RNA [32] (kindly provided by Dr. Arvind Patel (MRC, University of Glasgow Centre for Virus Research, University of Glasgow, Glasgow, UK)) were also maintained as above with the addition of puromycin (2 µg/mL) and nonessential amino acids (0.5 mL/50 mL). 

Infectious HCV virus was generated in the Huh-7.5 cell culture as previously described [28,29]. In brief, the pUC-JFH-1/AM7+1 plasmid kindly provided by Dr. A. Patel was linearized by Xba I, purified by a clean-up kit (Qiagen, Hombrechtikon, Switzerland), and used as a template for transcription using the TranscriptAid T7 High Yield Transcription Kit (Thermo Fischer Scientific, Waltham, MA, USA) according to the manufacturer’s protocol. The in-vitro transcribed genomic Jc1/JFH RNA purified by RNeasy Mini Kit (Qiagen) was used for electroporation of overnight grown Huh-7.5 cells. Viral stocks were obtained by harvesting the supernatants 72 h post electroporation, filtered, and aliquots were stored at −80 °C before use. TCID_50_ was determined by the Hierholzer and Killington method [59] using a plaque reduction assay as described below.

### 4.2. Cell Viability Assay

HEK293T, Huh-7.5, Huh7-J20, and Huh7-J17 cells were grown in a 96-well plate in the presence of tested compounds or DMSO for 48 or 72 h. Cell viability was analyzed by CellTiter 96 AQueous nonradioactive cell proliferation assay (MTS) (Promega, Madison, WI, USA) according to the manufacturer’s instructions. The cytotoxic concentration 50% (CC_50_) values were determined using CalcuSyn software (Biosoft, Cambridge, UK) from a dose response curves.

### 4.3. Drug Screening Assay

Huh-7.5 cells seeded in a 96-well plate were infected with HCVcc at MOI of 0.1. After 3 h, the virus was removed, fresh medium containing 50 μM of compounds or DMSO was added for 72 h, and IPMA assay to detect pseudo-plaques was performed. Cells were washed with PBS, fixed with chilled methanol, and permeabilized in 0.5% TritonX100 in PBS for 5 min. The cells were incubated with HCV anti-core (Hep C cAg antibody (C7-50); Santa Cruz Biotechnology, Dallas, TX, USA; 1:300 dilution) for 1 h followed by incubation with anti-mouse HRP-labelled secondary antibody (1:1000 dilution) for another 1 h. HCV pseudo-plaques were detected using a Vector Nova Red kit (Vector Laboratories Ltd., Peterborough, UK) and counted. 

For dose-response scales, Huh7-J20 cells were infected with HCVcc (MOI = 0.1) and treated for 72 h with various concentrations of compounds, DMSO or sofosbuvir. The levels of HCV infectivity and replication after inhibitory treatment were determined by the secreted alkaline phosphatase (SEAP) activity measurement using Phospha-Light kit (Applied Biosystems, Foster, CA, USA) according to the manufacturer’s instructions with minor modifications. In brief, 50 μL of culture supernatant was mixed with 50 μL of assay buffer and incubated for 7 min at room temp. Next, 50 μL of chemiluminescent substrate was added, incubated in the dark for 45 min, and the SEAP level was read in a luminometer. IC_50_ values were calculated using GraphPad Prism 6 software. 

For antiviral screening using the Huh7-J17 replicon cell line, cells were seeded together with various concentrations of compounds, DMSO or sofosbuvir. After 72 h, the influence on RNA replication was determined by measuring luciferase activity with the Bright-Glo Luciferase Assay system (Promega, Madison, WI, USA) according to the manufacturer’s instructions. IC_50_ values were calculated using GraphPad Prism 6 software.

### 4.4. HCV Pseudoparticle Assay 

HCVpp were generated as described previously [33]. Briefly, HEK293T cells were co-transfected with the retrovirus pMLV gag-pol vector, pMLV-Luciferase transfer vector, and expression plasmid encoding for HCV envelope glycoproteins (pCMV-E1E2). After 16 h post transfection the medium was collected and fresh medium with various concentrations of compounds, DMSO, or tunicamycin was added. Cell culture supernatants containing pseudoparticles were harvested 48 h post transfection and used to infect Huh-7.5 cells overnight. Infection efficiency after 72 h was measured by a Bright-Glo Luciferase Assay system (Promega, Madison, WI, USA). 

### 4.5. HCVpp Purification

After 48 h post transfection, as mentioned above, the medium from HEK293T cells containing HCVpp was collected and particles were purified by ultracentrifugation through 20% sucrose cushion in an SW41 Beckman rotor at 25,000 r.p.m. for 3 h. Pellets were suspended overnight in PBS to obtain 100-fold concentration. 

### 4.6. Time-of-Addition Assay 

Compounds were added at different time points during HCVpp production and infection of Huh-7.5 cells: before HEK2937 cell transfection (pre-treatment), during HEK 293T cell transfection for 72 h, before Huh-7.5 cell infection (pre-treatment), or during Huh-7.5 cell infection for 72 h. At each time point the infection efficiency was analyzed by luciferase measurement as described above. 

### 4.7. Western Blot Analysis 

HEK-293T cells were transfected with plasmid enabling the production of HCVpp. After 16 h post transfection the medium was collected and fresh medium containing different concentrations of uridine derivatives of 2-deoxy sugars, tunicamycin or DMSO was added. 48 post transfection cells were lysed with TNET buffer (20 mM Tris–HCl (pH 7.4), 150 mM NaCl, 1 mM EDTA, 1% Triton X-100) or HCVpp from the medium were purified by ultracentrifugation as mention above. Additionally, cell lysates were boiled in Glycoprotein Denaturing Buffer (0.5% SDS, 40 mM DTT) for 10 min, sodium phosphate (pH 7.5) and NP-40 were added to a final concentration of 50 mM and 1%, respectively, and samples were incubated for 5 h at 37 °C with or without PNGase F (New England Biolabs, Ipswich, MA, USA). Proteins were separated by SDS-PAGE under reducing conditions, transferred to PVDF membranes, and detected with specific mouse anti-E2 MAb (AP33) (diluted 1:2000), rat MLV Gag-specific MAb (1:300 dilution), or anti-actin (1:1000 dilution) antibody, followed by anti-rat alkaline phosphatase (AP)- or anti-mouse peroxidase (HRP)-conjugated secondary antibodies (diluted 1:2000). Antigen-antibody complexes were detected using a Super SignalWest Pico Substrate system (Pierce, Dallas, TX, USA) on the X-ray films (Fuji, Tokyo, Japan) or nitrotetrazolium blue (NBT) and 5-bromo-4-chloro-3-indolyl phosphate (BCIP) were used as substrates for alkaline phosphatase (AP).
